# ^18^F‐AV‐1451 uptake differs between dementia with lewy bodies and posterior cortical atrophy

**DOI:** 10.1002/mds.27603

**Published:** 2019-01-07

**Authors:** Zuzana Nedelska, Keith A. Josephs, Jonathan Graff‐Radford, Scott A. Przybelski, Timothy G. Lesnick, Bradley F. Boeve, Daniel A. Drubach, David S. Knopman, Ronald C. Petersen, Clifford R. Jack, Val J. Lowe, Jennifer L. Whitwell, Kejal Kantarci

**Affiliations:** ^1^ Department of Radiology Mayo Clinic Rochester Minnesota USA; ^2^ Department of Neurology Mayo Clinic Rochester Minnesota USA; ^3^ Department of Health Sciences Mayo Clinic Rochester Minnesota USA

**Keywords:** dementia with Lewy bodies, ^18^F‐AV‐1451, occipital association cortex, posterior cortical atrophy, tau PET

## Abstract

**Background:**

Posterior cortical atrophy and dementia with Lewy bodies are 2 distinct clinical syndromes, yet they can overlap in symptoms and occipital hypometabolism. Patients with dementia with Lewy bodies often have overlapping Alzheimer's disease pathology. Similarly, Lewy bodies can be found in patients with posterior cortical atrophy. We investigated differences in the distribution and magnitude of F18‐AV‐1451 uptake in patients with these 2 syndromes.

**Methods:**

Consecutive patients with probable dementia with Lewy bodies (n = 33), posterior cortical atrophy (n = 18), and cognitively unimpaired controls (n = 100) underwent ^18^F‐AV‐1451 positron emission tomography. Regional differences in AV‐1451 uptake were assessed using voxel‐wise and an atlas‐based approach. The greatest differences in AV‐1451 uptake between patient groups were identified using area under receiver operating curve statistics, and a composite region was derived.

**Results:**

AV‐1451 uptake in both patient groups was predominantly localized to the lateral occipital regions, but the magnitude of uptake was markedly greater in posterior cortical atrophy compared with dementia with Lewy bodies. The posterior cortical atrophy group showed the greatest AV‐1451 uptake throughout all the gray matter compared with that in other groups. The occipital composite region, consisting of superior, middle, and inferior occipital cortices, distinguished posterior cortical atrophy from dementia with Lewy bodies (area under the curve >0.97; *P* < 0.001, Bonferroni‐corrected) with excellent sensitivity (88%) and specificity (100%).

**Conclusions:**

Posterior cortical atrophy and dementia with Lewy bodies can share clinical features, and although the pattern of AV‐1451 uptake in occipital cortices overlaps between these 2 syndromes, its magnitude is significantly higher in posterior cortical atrophy. © 2019 The Authors. *Movement Disorders* published by Wiley Periodicals, Inc. on behalf of International Parkinson and Movement Disorder Society.

Patients with dementia with Lewy bodies (DLB) present with at least 1 of the typical clinical features such as rapid eye movement sleep behavior disorder (RBD), parkinsonism, fluctuating cognitive impairment, and recurrent visual hallucinations.^1^ Patients with posterior cortical atrophy (PCA) often present with a prominent and progressive decline in visual‐perceptual abilities such as simultanagnosia, optic ataxia, oculomotor apraxia, and dysgraphia.^2^ However, an overlap can exist between DLB and PCA in symptoms, imaging findings, and underlying pathologies. Both syndromes have been associated with hypometabolism on fluorodeoxyglucose (FDG) PET, localized to the occipital and temporoparietal cortices.^3^, ^4^, ^5^ Cingulate island sign on FDG PET was observed in both DLB and PCA, although it was more variable and asymmetric in PCA.^6^ Visual hallucinations, RBD, and parkinsonism can occur in PCA patients,^3^, ^7^, ^8^, ^9^, ^10^ and DLB patients can develop visuospatial difficulties.^11^ DLB patients often have Alzheimer's disease (AD) pathology in addition to Lewy bodies. Most PCA patients have AD, but in a subset of PCA patients, other pathologies were associated with the clinical syndrome, such as corticobasal degeneration, Creutzfeldt‐Jakob disease, or even Lewy body disease.^2^, ^12^

Novel imaging methods such as positron emission tomography with tau tracers may potentially have a diagnostic value in distinguishing the 2 disorders. The objective of this study was to compare the differences in the regional distribution and magnitude of the ^18^F‐AV‐1451 uptake on PET in DLB and PCA patients. We hypothesized that although DLB patients may show AV‐1451 uptake in occipital^13^, ^14^ and posterior temporoparietal cortical regions, similar in location to PCA patients,^15^, ^16^ the magnitude of AV‐1451 uptake would be higher in PCA patients.

## Methods

### Participants

Consecutively recruited patients with probable DLB (n = 33) were clinically examined by a behavioral neurologist, and underwent imaging examinations between May 2015 and May 2017 at the Mayo Clinic Alzheimer's Disease Research Center. Similarly, another sample of consecutive patients with PCA (n = 18) were clinically examined by a behavioral neurologist at the Mayo Clinic Department of Neurology, underwent imaging between August 2016 and October 2017, and were recruited into a National Institutes of Health‐funded study on atypical AD. Regardless of the recruitment strategy, all patients underwent a detailed examination by a behavioral neurologist, and diagnoses were rendered based on established clinical criteria. For comparison, we selected clinically unimpaired older adults (n = 100) similar in age to both the DLB and PCA patients from the Mayo Clinic Study of Aging, a population‐based study of aging.^17^ Each participant underwent neurological and neuropsychological evaluations and ^18^F‐AV‐1451 positron emission tomography (PET). Participants also had 11C‐Pittsburgh Compound B (PiB) PET and MRI at 3 T for anatomical labeling. Clinical evaluations and imaging visits were performed within a week of each other.

The study was approved by the Mayo Clinic Institutional Review Board. All participants and/or their proxies provided written consent to participate in this study.

PCA patients met clinical criteria for PCA.^2^, ^3^, ^9^ They had insidious disease onset with visual complaints in the absence of the primary ocular disease that would explain them, relatively preserved memory and insight into the early stages, progressive and disabling visual impairment, and any of the following symptoms: simultanagnosia with or without oculomotor apraxia or optic ataxia, visual field defects, constructional dyspraxia, or features of Gerstmann syndrome (agraphia, acalculia, finger agnosia, and left‐right disorientation). PCA patients were also evaluated for the presence of visual hallucinations, RBD, fluctuations, and parkinsonism.

DLB patients met the clinical criteria for probable DLB.^1^ The presence and severity of RBD, parkinsonism, visual hallucinations, and fluctuations were evaluated according to the 4th Consortium Criteria for DLB.^1^ Simultanagnosia was not specifically tested in DLB patients, and symptoms such as optic ataxia or oculomotor apraxia or elements of Gerstmann syndrome were evaluated during the clinical neurologic examination. Onset and duration of the specific features and dementia were ascertained during an interview by the attending physician with a patient and an informant.

Clinically unimpaired controls were independent functioning and performed within the normal range on tests covering 4 cognitive domains (executive, memory, language, visuospatial). The raw score from each test was transformed into an age‐adjusted score using established normative data from the Mayo Clinic's Older Americans Normative Studies^18^ on the participants from the same population. Participants scoring 1.0 SD or more than the age‐specific mean in the general population were considered to have possible cognitive impairment.^19^ The final decision on normal cognition was established during a consensus after taking into account visual or hearing deficits, education, and prior occupation and after reviewing all other information on the participant.

### PET Acquisition

Tau PET was performed using the ^18^F‐AV‐1451 tracer, and amyloid PET was performed using ^11^C‐PiB tracer on a PET/CT scanner (GE Healthcare; Milwaukee, WI) operating in 3‐D mode.^13^ For AV‐1451, an intravenous bolus injection of approximately 370 MBq (range, 330‐406 MBq) of AV‐1451 was administered, followed by an 80‐minute uptake period and a 20‐minute scan scan of four 5‐minute dynamic frames. For PiB, an injection of approximately 627 MBq (range, 384‐722 MBq) of PiB was administered, followed by a 40‐minute uptake period and a 20‐minute scan of four 5‐minute dynamic frames. AV‐1451 and PiB scans were visually inspected for technical quality including excessive motion by a trained PET technician.

For anatomical segmentation and labeling of PET images, a T1‐weighted 3‐dimensional high‐resolution magnetization‐prepared rapid‐acquisition gradient echo sequence was performed on a 3‐Tesla MRI scanner (General Electric Healthcare; Waukesha, WI) with an 8‐channel phased array head coil.

### PET Analysis

An automated image‐processing pipeline for PET image analysis included registration of the PET volumes to each patient's own T_1_‐weighted MRI for the segmentation of gray and white matter. Regional AV‐1451 uptake was calculated by transforming the Mayo Clinic Adult Lifespan Template (MCALT)^20^ with an in‐house modified 122‐region Automated Anatomic Labeling atlas^21^ into the native space of each T_1_‐weighted MRI using Advanced Normalization Tools software^22^ implemented in Statistical Parametric Mapping (SPM)12. MCALT is a publicly available population‐matched brain template (https://www.nitrc.org/projects/mcalt/).^23^ The Automated Anatomic Labeling (AAL) atlas has been modified to fit our MCALT template in SPM12. Fractions of tissue and cerebrospinal fluid (CSF) compartments in each PET voxel were derived from the registered T_1_‐weighted MRI. Correction for partial volume averaging of the CSF was performed using the 2‐compartment model.^24^ AV‐1451 uptake in each voxel was divided by the median value of the right and left cerebellar crus uptake.^25^ Cerebellar crus region corresponded to bilateral crus 1 and crus 2 in the AAL atlas.^21^

For PiB uptake, the global cortical retention standardized uptake value ratio (SUVR) was calculated from the bilateral parietal (including posterior cingulate and precuneus), orbitofrontal, prefrontal, temporal, and anterior cingulate regions, referenced to the right and left cerebellar crus.^26^

Group‐wise differences in magnitude of AV‐1451 uptake were assessed using a voxel‐wise and atlas‐based approach. Whereas the voxel‐wise analysis was exploratory to show the range and pattern of AV‐1451 uptake differences, especially between the PCA and DLB groups, the purpose of the atlas‐based analysis was to identify the region(s) with the greatest differences between PCA and DLB with a potential to differentiate between the 2 clinical groups. Comparisons were performed with and without correction for partial volume averaging of CSF. Because the findings were consistent between the 2 approaches, partial volume‐corrected findings are reported.

Voxel‐based differences in AV‐1451 uptake were assessed using statistical parametric mapping (SPM12). Maps showing the between‐group differences were displayed using a 2‐sided *t* test at *P* < 0.001 and cluster extent threshold >25.

Using the atlas‐based approach, we first calculated regional asymmetries in AV‐1451 uptake by subtracting right regional SUVR from left regional SUVR in 46 gray‐matter regions from the in‐house optimized AAL atlas and MCALT template described above. Because we did not identify any asymmetry between any 2 of the clinical groups, AV‐1451 uptake from the left‐ and right‐side regions was combined.

To identify gray‐matter regions with the greatest differences in AV‐1451 uptake between each two clinical groups, area under the curve (AUC) statistics were calculated for each individual gray‐matter region. Regions were ranked from highest to lowest AUC. Because multiple regions were compared, Bonferroni correction was used at *P <* 0.001. Our objective was to select regions with an area under the receiver operating curve (AUROC) above a certain threshold at *P* < 0.001 and Bonferroni‐corrected and to combine these into a “composite region” that would show the greatest differences in AV‐1451 uptake between the PCA and DLB groups.

### Statistics

Participants’ characteristics at the time of imaging were summarized using means and standard deviations (SD) or proportions (%). Continuous variables were compared across the groups using analysis of variance, followed by a priori chosen contrasts for pair‐wise comparisons of the DLB versus PCA groups. Categorical variables were compared across the groups using the chi‐square test or logistic regression between the PCA and DLB groups. Statistics along with AUROC sensitivity and specificity were calculated to measure the accuracy of the AV‐1451 uptake in a composite region to distinguish between the DLB and PCA groups. The same calculation was performed for the global cortical PiB SUVR.

Statistical analyses were performed using SAS version 9.4 (SAS Institute, Cary, NC) and R statistical software version 3.1.1 (R project.org).

## Results

### Participants’ Characteristics

Demographic, clinical, and basic imaging characteristics at the time of imaging are summarized in Table 1. Eighty‐five percent of DLB patients were men, whereas 61% of PCA patients were women. Controls were similar in age to those the DLB and PCA groups and did not differ in sex distribution from either patient group. Simultanagnosia, optic ataxia, ocular apraxia, Gerstmann syndrome, visual field deficits, and hemineglect were more frequent in PCA patients. Similarly, RBD, parkinsonism, visual hallucinations, and fluctuations were more frequent in patients with DLB. Symptoms typical for PCA were not manifested in DLB patients on neurological examination.

**Table 1:** Participants’ characteristics

	CN n = 100	DLB n = 33	PCA n = 18	Overall *P* a	DLB vs PCA *P* b
Age, years	66.9 (9.7)	68.1 (7.8)	64.6 (6.6)	0.42	0.19
Male, n (%)	68 (68%)	28 (85%)	7 (39%)	0.003	< 0.001
CDR sum of boxes	0.0 (0.2)	4.8 (3.6)	5.4 (3.6)	< 0.001	0.29
MMSE	28.9 (1.0)	24.0 (5.2)	NA	< 0.001	
MoCA	NA	17.8 (7.0)	17.2 (6.3)		0.80
Cortical PiB SUVR	1.36 (0.07)	1.69 (0.45)	2.36 (0.39)	< 0.001	< 0.001
Occipital composite AV‐1451 SUVR	1.13 (0.08)	1.20 (0.10)	2.49 (0.64)	< 0.001	< 0.001
Occipital composite AV‐1451 SUVR range (min, max)	0.89, 1.33	1.01, 1.42	1.31, 3.77		
Visual hallucinations, n (%)	NA	13 (39%)	2 (11%)^c^		
Fluctuations, n (%)	NA	22 (67%)	1 (6%)^c^		
Parkinsonism, n (%)	NA	30 (91%)	1(6%)^c^		
RBD, n (%)	NA	31 (94%)	4 (22%)^c^		
Simultanagnosia, n (%)	NA	NA	15 (83%)		
Optic ataxia, n (%)	NA	0	7 (39%)		
Oculomotor apraxia, n (%)	NA	0	8 (44%)		
Gerstmann syndrome, n (%)	NA	0	10 (56%)		
Visual field deficits, n (%)	NA	0	4 (25%)^d^		
Hemineglect, n (%)	NA	0	1(6%)^d^		

a *P* values for differences between groups come from an ANOVA for the continuous variables or a chi‐square test for the categorical variables.

b *P* values for differences between groups are contrasts from an ANOVA for the continuous variables or logistic regression for the categorical variables.

c First PCA patient with RBD also had visual hallucinations and parkinsonism. Second PCA patient with RDB also had visual hallucinations. Third PCA patient with RBD also had fluctuations. Fourth PCA patient had isolated RBD without other DLB clinical features.

d Information on visual field deficits and hemineglect was not available for 2 PCA patients.

CN, cognitively unimpaired adults; DLB, dementia with Lewy bodies; PCA, posterior cortical atrophy; SUVR, standardized uptake value ratio; CDR‐SOB, Clinical Dementia Rating, sum of boxes; MMSE, Mini Mental State Examination; MoCA, Montreal Cognitive Assessment; RBD, REM sleep behavior disorder.

The features presented in the table are related to image acquisition and not to the time of disease onset.

Four PCA patients had clinically described sleep disorder that was consistent with RBD at the time of imaging (Table 1). One of these 4 PCA patients had isolated RBD. One PCA patient had RBD and visual hallucinations. One PCA patient had RBD, visual hallucinations, and also mild parkinsonism. One had RBD and fluctuations. These PCA patients would also meet the clinical criteria for possible or even probable DLB. Additional DLB clinical features in patients with PCA were present at the imaging visit. However, all 4 PCA patients with additional DLB clinical features initially presented with typical PCA symptoms, not with DLB symptoms, and were diagnosed as having PCA not DLB.

All PCA patients had elevated PiB uptake (mean PiB SUVR ± SD, 2.36 ± 0.39). However, 2 patients had lower PiB SUVR (1.51 and 1.56) compared with other PCA patients. These 2 also had the lowest AV‐1451 uptake among all PCA patients, supporting the notion that there might be other etiologies contributing to clinical symptoms in addition to their mild AD pathology. These 2 PCA patients were carefully evaluated during multiple visits, and no other clinical features suggesting, for example, corticobasal syndrome, Creutzfeldt‐Jakob disease, or other known etiology were observed. For comparison, mean PiB SUVR ± SD was 1.69 ± 0.45 in DLB patients and 1.36 ± 0.07 in controls.

### Voxel‐Wise Differences in AV‐1451 Uptake

**Figure 1:**
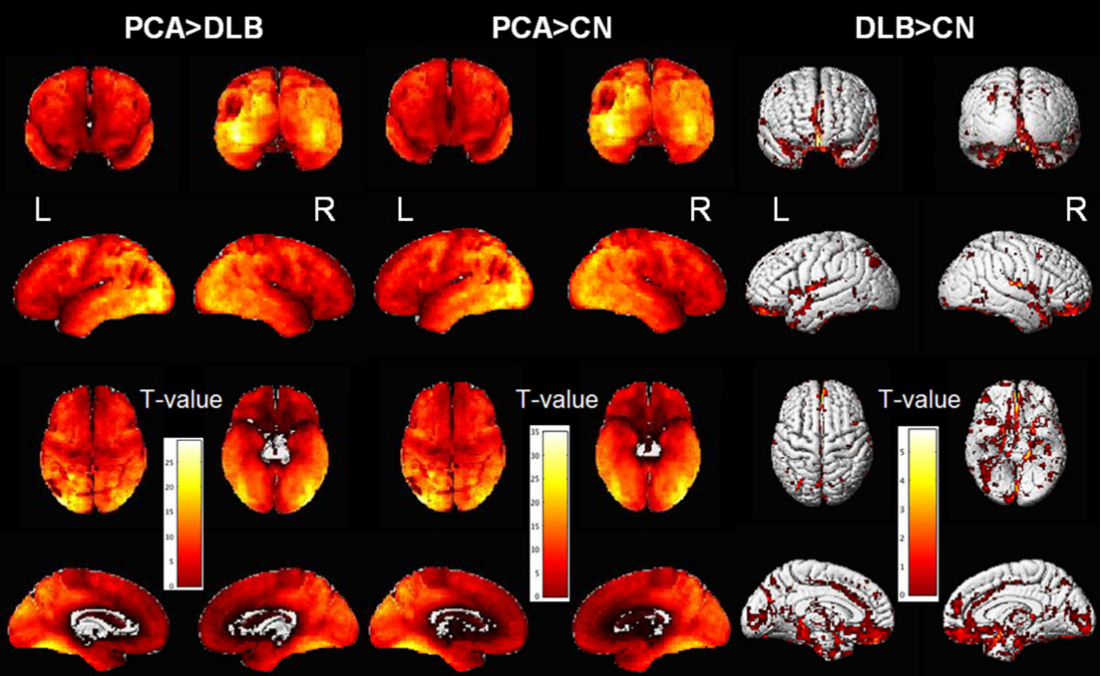
Voxel‐wise differences in AV‐1451 uptake. The surface renderings show regional differences in AV‐1451 uptake between the clinical groups.

Figure 1 illustrates the pattern of regional differences in cortical AV‐1451 uptake between each two clinical groups. PCA patients had markedly higher AV‐1451 uptake across the entire cortex compared with DLB patients, with the greatest differences in the lateral occipital association cortices: superior, middle and inferior occipital gyri. No regions showed higher AV‐1451 uptake in DLB patients compared with PCA patients. As expected, PCA patients showed markedly higher AV‐1451 uptake across the entire cortex compared with controls. DLB patients showed only modest differences in AV‐1451 uptake compared with controls, scattered in temporal, parietal, occipital, and also frontal cortices.

### Atlas‐Based Differences in Magnitude of AV‐1451 Uptake

**Figure 2:**
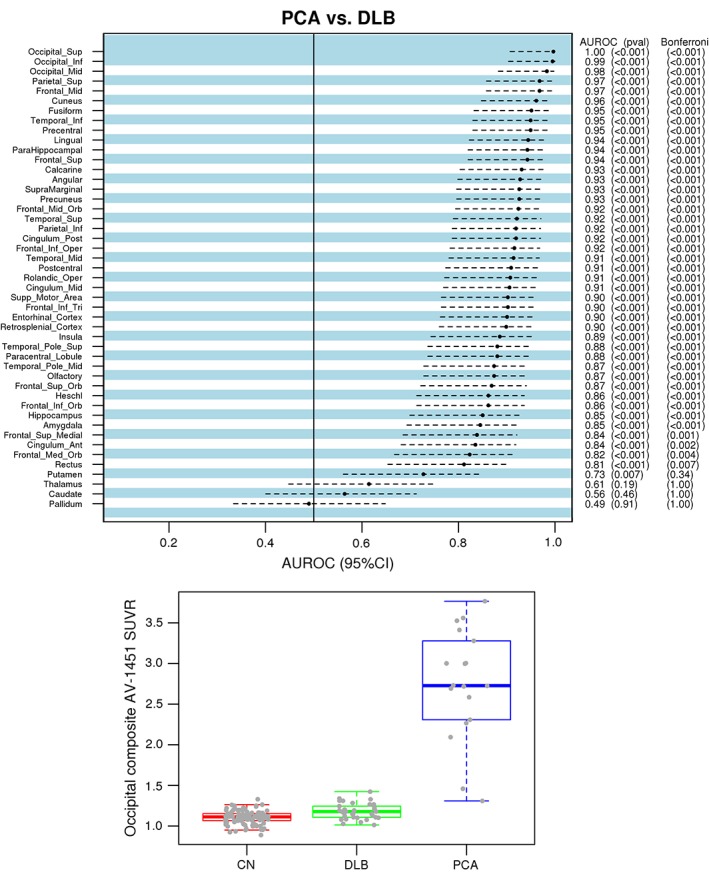
Atlas‐based analysis for the differences in AV‐1451 uptake among DLB and PCA groups. Regional differences in AV‐1451 uptake between the PCA and DLB groups are plotted across 46 gray‐matter regions and ranked using area under curve (AUC) statistics (top). Regions with AUC > 0.97 are combined into the occipital composite (occipital superior, middle, and inferior cortices). Box‐and‐whiskers plots below show differences in occipital composite AV‐1451 uptake by clinical group (bottom).

Figure 2 (top) shows the differences in AV‐1451 uptake between the PCA and DLB groups, expressed as AUC for the individual gray‐matter regions. AUCs are ranked from the highest to the lowest. The highest‐ranking regions with AUC > 0.97 were lateral occipital association cortices (superior, middle, and inferior occipital), and these regions were combined into the occipital composite, taking the median SUVR from the entire composite (overall AUC > 0.97). Although the cutoff AUC at >0.97 was arbitrary, the occipital regions selected into this composite were consistent with regions showing the greatest differences in AV‐1451 uptake between PCA and DLB in our voxel‐wise analysis.

Figure 2 (bottom) shows that the AV‐1451 uptake in the occipital composite in PCA (mean SUVR ± SD, 2.75 ± 0.68) was significantly higher than in DLB (mean SUVR ± SD, 1.18 ± 0.10; *P* < 0.001) and controls (mean SUVR ± SD, 1.10 ± 0.08; *P* < 0.001). Among the 4 PCA patients who had additional DLB clinical feature(s), the lowest occipital composite AV‐1451 SUVR was 2.26, whereas among all DLB patients, the highest occipital composite AV‐1451 SUVR was 1.42. Therefore, all DLB patients could be separated from PCA patients with additional DLB features by the occipital composite AV‐1451 SUVR.

**Figure 3:**
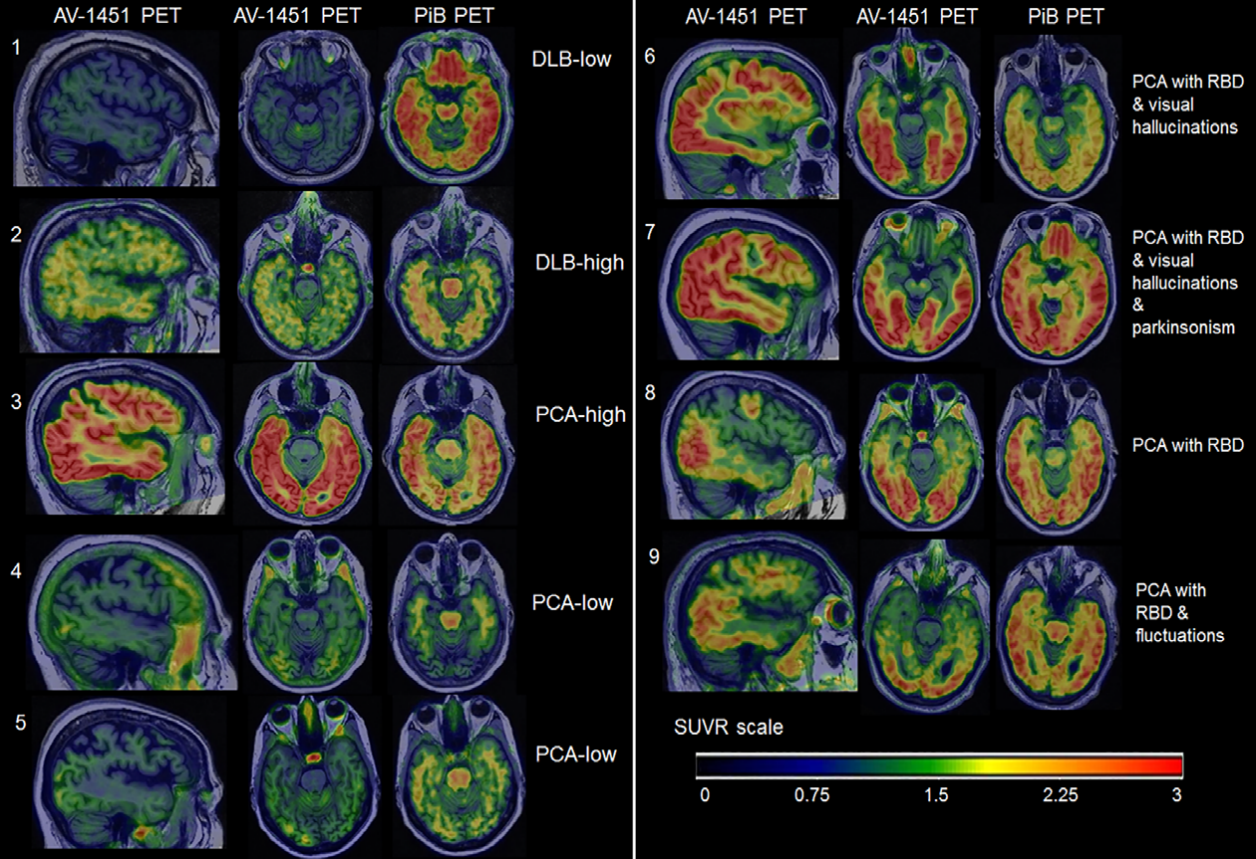
Examples of PET images in individual patients. Individual AV‐1451 scans show focal AV‐1451 uptake and PiB uptake. 1. *DLB with low AV‐1451 SUVR* is a 78‐year‐old right‐handed man. As expected, he has low occipital composite AV‐1451 SUVR of 1.02 and global PiB SUVR of 1.45. He has parkinsonism, RBD, visual hallucinations, and fluctuations. 2. *DLB with high AV‐1451 SUVR* is an 82‐year‐old right‐handed man with high occipital composite AV‐1451 SUVR of 1.42 compared with other DLB patients in the study. Global PiB SUVR is 1.46. He has parkinsonism and RBD but no visual hallucinations or fluctuations. 3. *PCA with high AV‐1451 SUVR* is a 59‐year‐old right‐handed man with high occipital composite AV‐1451 SUVR of 3.76 and global PiB SUVR of 2.52. He presented with profound visuospatial deficits, severe simultanagnosia, and also mild problems with memory. He does not have RBD, parkinsonism, visual hallucinations, or fluctuations. 4. *PCA with low AV‐1451 SUVR* is a 76‐year‐old right‐handed woman with low occipital composite AV‐1451 SUVR of 1.45. Global PiB SUVR is also low at 1.56 compared with other PCA patients. She presented with a primary progressive alexia and severe visuoperceptual impairment. She did not have any DLB features or features suggestive of corticobasal syndrome, frontotemporal dementia, or Creutzfeldt‐Jakob disease. 5. *PCA with low AV‐1451 SUVR* is a 73‐year‐old right‐handed woman with occipital composite AV‐1451 SUVR of 1.31 and global PiB SUVR of 1.51, with severe Gerstmann syndrome, constructional dyspraxia, and mild executive weakness and simultanagnosia. 6. *PCA with RBD and visual hallucinations* is a 57‐year‐old left‐handed woman with occipital composite AV‐1451 SUVR of 3.00 and global PiB SUVR of 2.02, with simultanagnosia, optic ataxia, oculomotor apraxia, and partial Gerstmann syndrome. 7. *PCA with RBD, visual hallucinations, and parkinsonism* is a 58‐year‐old right‐handed woman with occipital composite AV‐1451 SUVR of 3.27 and global PiB SUVR of 2.79, with simultanagnosia, optic ataxia, oculomotor apraxia, and Gerstmann syndrome. 8. *PCA with RBD* is a 71‐year‐old right‐handed man with occipital composite AV‐1451 SUVR of 2.72 and global PiB SUVR of 2.45. He has severe visuospatial impairment and simultanagnosia. 9. *PCA with RBD and fluctuations* is a 68‐year‐old right‐handed man with occipital composite AV‐1451 of 2.26 and global PiB SUVR of 2.48. He presented with quadrantanopia, progressive aphasia, and working memory problems.

Figure 3 shows imaging findings that were: (1) typical within the DLB and PCA groups, (2) less typical in the 2 PCA patients with low PiB uptake and low occipital composite AV‐1451 uptake, and (3) all 4 PCA patients with additional DLB clinical features.

### Distinguishing DLB and PCA Patients Using AV‐1451 Uptake in the Occipital Composite Region

**Figure 4:**
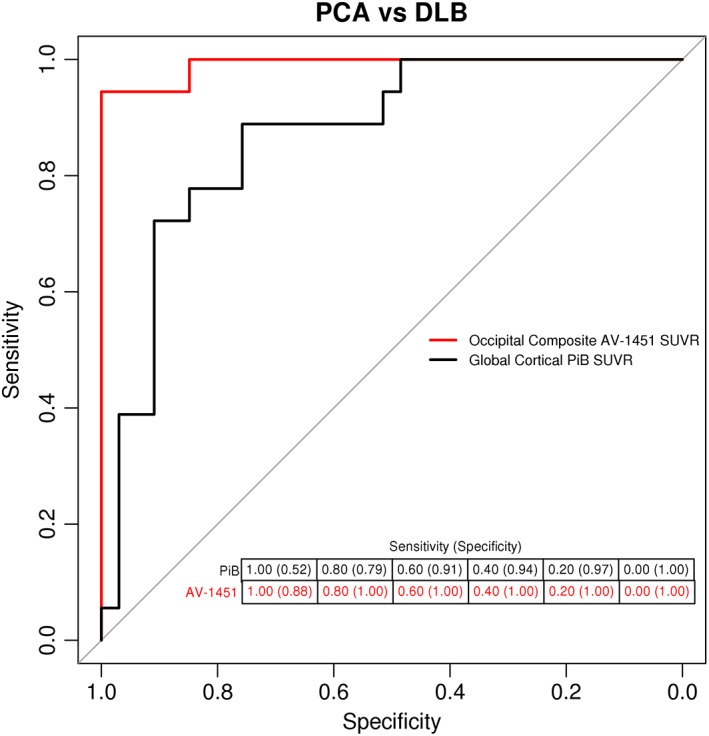
Discrimination between PCA and DLB using the occipital composite region AV‐1451 uptake. Discrimination between DLB and PCA patients using AV‐1451 uptake from the occipital composite. For comparison, cortical PiB uptake is used. The accuracy of the occipital composite AV‐1451 uptake to distinguish between DLB and PCA patients corresponds to area under the receiver operating curve of 0.97. The accuracy of the cortical PiB uptake to distinguish between groups corresponds to area under the receiver operating curve of 0.87.

Figure 4 shows the discrimination between the PCA and DLB groups using the receiver operating characteristics curves with associated AUROC for the occipital composite AV‐1451 SUVR. Because elevated PiB uptake is common in PCA patients and also in many DLB patients, we included global PiB SUVR as a comparison for distinguishing among the 2 clinical groups. The accuracy of occipital composite AV‐1451 uptake in distinguishing the PCA from DLB group was >0.97, whereas the accuracy of cortical PiB SUVR was 0.87. At various step‐wise sensitivity levels (Fig. 4), the specificity of the occipital composite AV‐1451 was higher compared with the global cortical PiB SUVR.

## Discussion

We compared AV‐1451 uptake in PCA and probable DLB patients from 2 consecutive samples diagnosed using the standard clinical diagnostic criteria for both syndromes. For comparison, cognitively unimpaired controls were selected from a population‐based sample. We demonstrated that PCA patients had markedly higher AV‐1451 uptake on PET across the entire cortex compared with DLB patients and controls. In PCA patients, AV‐1451 uptake was higher especially in the lateral occipital association cortices compared with DLB patients. Based on the voxel‐based and subsequent atlas‐based analysis, we identified the occipital composite cortical region with the greatest difference in AV‐1451 uptake between PCA and DLB patients. Four PCA patients had DLB features in addition to cardinal PCA features at the time of imaging. The AV‐1451 uptake in the occipital composite was higher in all 4 PCA patients compared with any DLB patient. In the discrimination analysis using AUROC, the occipital composite AV‐1451 uptake distinguished PCA from DLB patients with excellent sensitivity (88%) and specificity (100%). Overall, AV‐1451 PET imaging showed significant differences between DLB and PCA patients, including the occasional PCA patients who had DLB clinical features overlapping with PCA features.

The pattern of AV‐1451 uptake in DLB patients^13^, ^14^, ^27^ and PCA^15^, ^16^, ^28^ compared with cognitively unimpaired controls has been described by us and other groups**,** with an uptake in the bilateral occipital and to some extent also posterior temporoparietal cortices in both syndromes, and significant uptake in the lateral temporal and frontal cortices in PCA. In the current study, we compared differences in AV‐1451 uptake between PCA and DLB patients because these 2 syndromes can overlap in clinical symptoms and imaging findings.

We found substantially higher AV‐1451 uptake throughout the entire cortex in PCA patients compared with DLB, with findings consistent between the voxel‐wise and atlas‐based analyses. The greatest differences between DLB and PCA patients were seen in the lateral occipital association cortices, combined into the occipital composite region. Except for the 2 PCA patients with relatively low AV‐1451 and low PiB uptake, the separation between DLB and PCA groups was complete with the occipital composite AV‐1451 uptake.

Although the differences in AV‐1451 uptake were expected, the magnitude of these differences was significant. High AV‐1451 uptake in our PCA patients agrees with the notion that AD pathology is the most frequent cause of PCA.^2^, ^9^, ^29^ On the other hand, many DLB patients also have varying degrees of AD pathology in addition to Lewy body disease.^30^, ^31^ Despite this overlap, the differences in AV‐1451 uptake between PCA and DLB were substantial. We included a consecutive sample for both PCA and DLB patients to account for various possible underlying etiologies, especially in PCA patients. However, all PCA patients from the current sample had elevated PiB and AV‐1451 uptake levels. In 2 PCA patients with lower, although still elevated**,** levels of global PiB SUVR and the occipital composite AV‐1451 SUVR, we did not identify any other underlying etiologies based on available clinical data.

AV‐1451 tracer binds to the same tau strains in PCA and DLB patients.^32^ However, the AV‐1451 tracer does not completely reflect early‐stage tau progression suggested by Braak neurofibrillary tangle staging, particularly in atypical forms of AD. Both PCA and DLB have shown atypical patterns of tau deposition that do not fit the traditional Braak staging scheme.^33^, ^34^ Should other pathologies be present in our PCA patients, such as TAR DNA‐binding protein‐43 or corticobasal degeneration, they would likely diminish the differences in AV‐1451 uptake between PCA and DLB but not vice versa.

Interestingly, 4 PCA patients had overlapping RBD alone or in combination with other DLB clinical features at the time of imaging. However, the AV‐1451 uptake in the occipital composite was still significantly higher in all 4 of these PCA patients with additional DLB features than in any probable DLB patient. DLB features in these 4 PCA patients could be explained by the presence of Lewy body pathology in addition to AD pathology.^35^ RBD is strongly suggestive of α‐synucleinopathy.^36^ However, patients with presumed or definite AD rarely can also have RBD and other DLB symptoms^37^ as a result of AD pathology with neuronal loss in the vulnerable regions such as the substantia nigra. However, the definite substrate underlying RBD in these rare cases remains unclear.^38^, ^39^ On the other hand, co‐occurrence of Lewy body pathology in addition to dominant AD pathology was the least frequent in hippocampal‐sparing AD cases compared with typical AD and limbic‐predominant cases in a large autopsy‐confirmed cohort.^33^ Clinical PCA would be the most consistent with autopsy‐confirmed hippocampal‐sparing AD. Overall, our PCA patients with additional DLB clinical features likely have AD as the primary underlying etiology of PCA, and it is also possible they have Lewy body disease in addition to AD.

The overall accuracy of occipital composite AV‐1451 uptake in distinguishing between PCA and DLB patients was excellent, with 100% specificity at a sensitivity of 88%. This finding can be useful in the differential diagnosis and management of PCA and DLB patients. As expected, accuracy and specificity of global cortical PiB uptake were lower because many DLB patients have elevated uptake on PiB PET.^40^

A limitation of the current study is that polysomnography was not routinely performed in all PCA patients who had REM sleep behavior disorder symptoms, and RBD was recorded based on clinical evaluation. Another limitation is that not all specific PCA‐related symptoms were measured in all patients, that is, simultanagnosia was not measured in DLB patients. Finally, we cannot exclude the possibility that regional differences in AV‐1451 uptake were contributed by different stages of clinical progression in the PCA and DLB groups. Although our DLB and PCA groups did not differ on Montreal Cognitive Assessment or Clinical Dementia Rating, sum of boxes scales, these measures assessing the global clinical severity are perhaps not the most optimal measures to compare the severity of the 2 clinical syndromes. Despite these limitations, the striking differences in AV‐1451 uptake between DLB and PCA patients demonstrate the diagnostic value of AV‐1451 PET imaging in these 2 syndromes with occasionally overlapping symptoms.

## Author Roles

1) Research project

A. Conception: Zuzana Nedelska, Kejal Kantarci

B. Organization: Zuzana Nedelska, Keith A. Josephs, Val J. Lowe, Ronald C. Petersen, Clifford R. Jack, Jr., Jennifer L. Whitwell, Kejal Kantarci

C. Execution: Zuzana Nedelska, Keith A. Josephs, Jonathan Graff‐Radford, Bradley F. Boeve, Daniel A. Drubach, David S. Knopman

2) Statistical Analysis

A. Design: Scott. A. Przybelski, Timothy G. Lesnick, Zuzana Nedelska

B. Execution: Scott. A. Przybelski, Timothy G. Lesnick

C. Review and Critique: Scott. A. Przybelski, Timothy G. Lesnick, Zuzana Nedelska, Kejal Kantarci

3) Manuscript

A. Writing of the first draft: Zuzana Nedelska

B. Review and Critique: Zuzana Nedelska, Keith A. Josephs, Jonathan Graff‐Radford, Scott. A. Przybelski, Timothy G. Lesnick, Bradley F. Boeve, Daniel A. Drubach, David S. Knopman, Ronald C. Petersen, Clifford R. Jack, Jr., Val J. Lowe, Jennifer L. Whitwell, Kejal Kantarci

## Disclosures outside this work

Dr. Graff‐Radford receives research support from the NIH (K76‐AG057015). Dr. Josephs receives research support from the NIH (R01‐AG37491, R01‐NS89757, R01‐DC14942, R01‐DC12519). Dr. Boeve has served as an investigator for clinical trials sponsored by GE Healthcare and Axovant; receives royalties from the publication of a book titled *Behavioral Neurology of Dementia* (Cambridge Medicine, 20179); serves on the Scientific Advisory Board of the Tau Consortium; receives research support from the NIH (U01‐AG045390, U54‐NS092089, R01‐AG041797, U01‐NS100620, R01‐AG38791), the Mayo Clinic Dorothy and Harry T. Mangurian Jr. Lewy Body Dementia Program, and the Little Family Foundation.

Dr. Knopman served as Deputy Editor for *Neurology*; served on a Data Safety Monitoring Board for Lilly Pharmaceuticals; serves on a Data Safety Monitoring Board for Lundbeck Pharmaceuticals and for the DIAN study; served as a consultant to TauRx Pharmaceuticals ending in November 2012; was an investigator in clinical trials sponsored by Baxter and Elan Pharmaceuticals in the past 2 years; is currently an investigator in a clinical trial sponsored by TauRx; and receives research support from the NIH (U01‐HL096917, AG‐037551). Dr. Petersen consults for Roche, Inc., Merck, Inc., Genetech, Inc., Biogen, Inc., and Eli Lilly and Company, Pfizer, Elan Pharmaceuticals, Wyeth Pharmaceuticals, and GE Healthcare, receives royalties from the publication of *Mild Cognitive Impairment* (Oxford University Press, 2003), and receives research support from the NIH (UF1‐AG32438, U19‐AG24904, RF1‐AG57547, U01‐AG016976).

Dr. Lowe consults for Bayer Schering Pharma, Piramal Life Sciences and receives grants from GE Healthcare, Siemens Molecular Imaging, and AVID Radiopharmaceuticals, the MN Partnership for Biotechnology and Medical Genomics, and the Leukemia & Lymphoma Society, and receives research support from the NIH (R01CA‐200551, P50‐CA102701, UM1CA‐186686, P50‐AG44170, R01‐DC012519, R01CA‐154348, R01‐NS 89757). Dr. Jack consults for Eli Lilly and serves on an independent data‐monitoring board for Roche but he receives no personal compensation from any commercial entity; he receives research support from the NIH (UF1‐AG32438, U19‐AG24904, R01‐AG46179, R01‐AG037551). Dr. Whitwell receives research support from the NIH (R01‐DC12519, R01‐NS89757, R01‐AG37491, and R01‐DC14942). Dr. Kantarci s serves on the data safety‐monitoring board for Takeda Global Research and Development Center, Inc.; receives research support from Avid Radiopharmaceuticals and Eli Lilly, and receives funding from NIH (P50‐AG044170‐01‐ Project 2, P50‐AG16574, UF1‐AG32438, K12‐HD65987, U19‐AG24904, U01‐AG45390, R01‐AG46179, RF1‐AG51504, U01‐AG52943, RF1‐AG55151, R01‐AG38791, RF1‐AG57547, R01‐AG55121, R01‐NS80816), Bluefield Project to Cure Frontotemporal Dementia (AB‐BFP‐2016), NCATS (KL2TR 02379), and Alzheimer's Drug Discovery Foundation (2013646).
